# Frequency jumps and subharmonic components in calls of female *Odorrana tormota* differentially affect the vocal behaviors of male frogs

**DOI:** 10.1186/s12983-023-00517-9

**Published:** 2023-12-08

**Authors:** Yatao Wu, Xiuli Luo, Pan Chen, Fang Zhang

**Affiliations:** 1 College of Life Sciences, Anhui Normal University, No. 1 Beijing East Road, Wuhu, 241000 Anhui China; 2 Anhui Provincial Key Laboratory of the Conservation and Exploitation of Biological Resources, Anhui Normal University, Wuhu, 241000 Anhui China

**Keywords:** *O. tormota*, Nonlinear phenomena, Vocal behaviors, Playback experiments

## Abstract

Studies have demonstrated that the sounds of animals from many taxa with nonlinear phenomena (NLP)—caused by nonlinear characteristics of vocal organ dynamics that lead to nonlinear vocal phenomena—can influence the behavior of receivers. However, the specific functions of different NLP components have received less attention. In most frog species, females produce few or no vocalizations; in contrast, female *Odorrana tormota* exhibit a diverse range of calls that are rich in NLP components. Previous field playbacks have shown that the female calls can elicit responses from male frogs. Therefore, we conducted a phonotaxis experiment to investigate the differential effects of different NLP calls by female *O. tormota* on the vocal behavior of male frogs. The results revealed that calls with subharmonics elicited a greater number of short calls and answering calls from male frogs compared to calls with frequency jumps. However, calls with frequency jumps triggered more staccato calls from males than calls with subharmonics. Additionally, during the phonotaxis experiments, we recorded the initial vocalizations of males in response to playbacks of female calls. The majority of males first produced short calls. Under calls with frequency jumps, most of male frogs approaching within 10 cm of the loudspeaker produced staccato calls instead of “meow” calls or short calls. While under calls with subharmonics, most male frogs preferred to produced short calls. Our findings demonstrate that frequency jumps and subharmonic components in the calls of female *O. tormota* have different effects on male vocal behaviors. The current study lays a foundation for a further understanding of the function of anuran NLP components.

**Supplementary Information:**

The online version contains supplementary material available at 10.1186/s12983-023-00517-9.

## Background

In bioacoustics, nonlinear phenomena (NLP) are commonly measured in terms of frequency jumps (an abrupt change in the fundamental frequency (*f*_*0*_) within 1 ms, Fj), subharmonics (existing at 1/*n* of *f*_*0*_, Sh), deterministic chaos (in which the energy is diffusely distributed, Ch), and biphonation (calls containing at least two bifurcations in *f*_*0*_ or subharmonics, Bp) [1]. Mechanistically, NLP are caused by nonlinear characteristics of vocal organ dynamics (e.g., differences in size, thickness, tension between the two sides of the vocal folds) that lead to nonlinear vocal phenomena [1, 2]. Currently, NLP are observed in the calls of many animal taxa [3–9] and even in cries of newborn human infants [10]. Compared with harmonic calls, nonlinear calls have larger frequency fluctuations, more complex structures, and diverse forms, which increase both call complexity and individual specificity [1, 11, 12]. Many studies have reported that NLP play an important role in the communication of some animals, such as preventing habituation [13], making calls more prominent [14, 15], encoding individual information (such as health status, e.g., [1]; body length, e.g., [13]), attracting attention from receivers [6, 15], increasing sexual attraction [16, 17], and even affecting the transmission of human emotions [18]. However, functional studies of NLP have focused on individual recognition, alerting and defense; less attention has been given to the role of NLP in courtship, especially in anurans [17], which heavily rely on sound for reproductive behavior.

Researchers have found that male and female concave-eared torrent frogs (*Odorrana tormota*), a nocturnal species that inhabits vegetation along noisy streams, produce courtship calls with ultrasonic and NLP components [4, 19]. A total of 39% of female calls and 93% of male calls contain at least one NLP; male calls include long calls, short calls, staccato calls, and “meow” calls [3, 4, 20]. These high rates suggesting that NLP are not accidental but rather important components of courtship calls in this species. Anurans display sex differences in calling behavior [21]. Typically, females do not engage in courtship calls during the breeding season, and those that vocalize possess only a rudimentary larynx and emit feeble reciprocal calls [22], rapping sounds [23], or simple releasing calls [21, 24]. Female concave-eared torrent frogs are an exception, demonstrating high-frequency and moderately intense mating calls. Shen [25] carried out acoustic playback experiments in the field and in a quiet room and found that male concave-eared torrent frogs rapidly and precisely approached the loudspeakers in response to female calls. Zhang [4] suggested that NLP components in female calls can serve as individual signatures. Recent studies have also suggested that NLP components in calls of male *O. tormota* increase sexual attractiveness [17]. Taken together, these findings suggest that calls play an important role in bidirectional male–female communication among concave-eared torrent frogs, but the effect of female NLP components on the vocal behaviors of male frogs remains unclear.

In the present study, we categorized the calls of female concave-eared torrent frogs and found that calls containing a single type of NLP component (frequency jumps or subharmonics) accounted for more than 50% of all calls containing NLP components. The remaining calls containing NLP had at least two types of NLP components. Therefore, the purpose of the present study was to investigate whether frequency jumps and subharmonics in the calls of female frogs differentially affect the vocal behaviors of male frogs.

## Materials and methods

### Study site

A field study was performed in the mountain range of Huangshan (Anhui Province, China) in the village of Fuxi along Fu Creek (118° 08′ 44.89″ E, 30° 05′ 01.61″ N, elevation: 600 m a.s.l.), in March and April of 2021, 2022 and 2023. During the breeding period, the male concave-eared torrent frogs usually appeared at approximately 16:00 and called from rocks or vegetation on the banks of the stream, and at 22:00, the calling behavior of male frogs has mostly ceased. During the study period (18:00–23:00), the nightly ambient temperature at the study site fluctuated widely, but the temperature and humidity at which males were observed in the field ranged from 15.0 to 20.0 °C and from 85 to 95%, respectively.

### Sound recordings

All female calls were recorded using a sound recorder (Sound Devices 702, WI, USA; frequency range: 10 Hz–96 kHz) and an AKG microphone (AKG model C417, Vienna, Austria) with the microphone placed approximately 5–10 cm above the female frog’s head. Recordings were carried out during the peak activity period (mainly between 19:00 and 23:00) in the breeding season (March and April) under similar ambient conditions (temperature: 15–20 °C; humidity: 80–99%; ambient noise: 65–75 dB SPL).

### Phonotaxis experiment

**Fig. 1:**
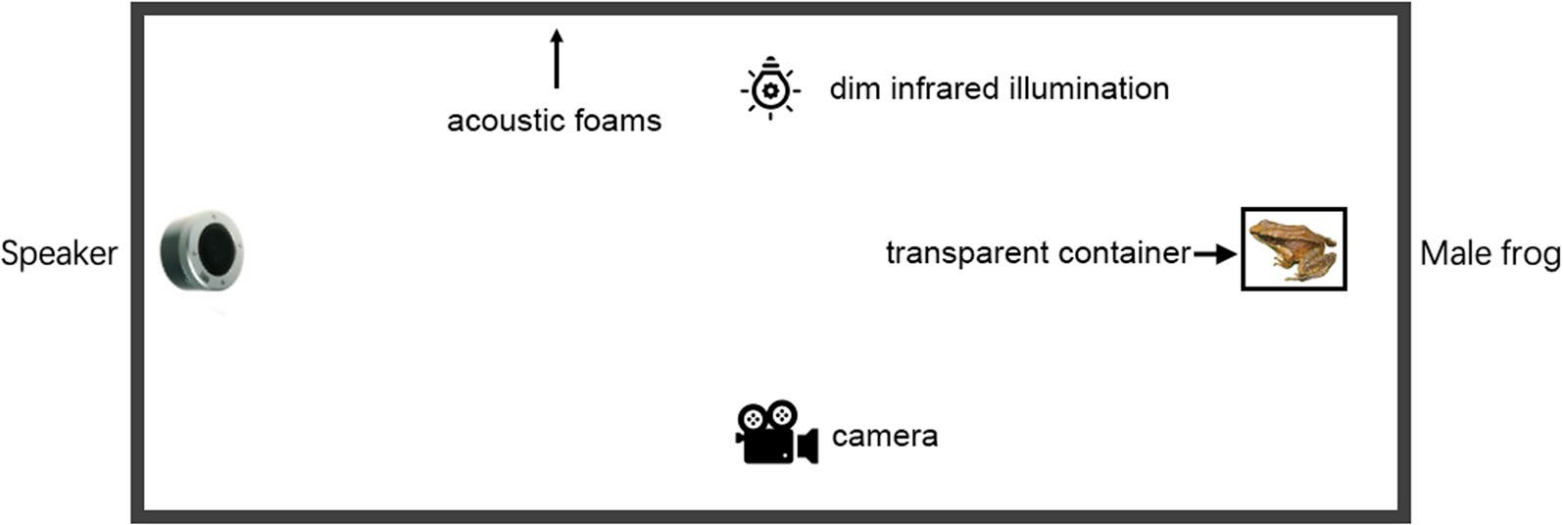
Schematic diagram of the phonotaxis experiment (L × W × H: 170 × 120 × 150 cm)

A total of 120 male frogs were captured in the field and placed in individual plastic terraria for the playback experiment. We used acoustic foam (thickness: 180 mm) to construct a platform (L × W × H: 170 × 120 × 150 cm) in a dark and quiet room (Fig. 1) approximately 1.2 km from the frog’s natural habitat; phonotaxis tests were conducted at ambient temperature and humidity levels of ~ 17 °C and ~ 85%, respectively. To simulate the natural habitat, we collected pebbles from the concave-eared torrent frog’s habitat and placed them inside the playback platform. Additionally, we used a spray bottle to mist the platform with stream water, keeping the ground moist. To facilitate determination of instances in which the male frogs approached within a 10 cm range of the loudspeaker, we placed a white adhesive tape around the speaker at a distance of 10 cm. A dim infrared light source was placed 1.5 m directly above the center of the playback experiment platform [25]. To investigate the effect of a single NLP component of female frogs on male amplexus behavior, and to prevent false positives in the experimental results [26], we selected 12 stimulus calls (see Additional file 1: Fig. S1); of these, six contained only frequency jump components, while the remaining ones consisted solely of subharmonic components. These calls were obtained from six female frogs. During the experiment, a stimulus (one of the 12 playback calls) was played intermittently through a loudspeaker (HP SS10 BT Speaker, Shanghai, China) at the rate of 1 call per 15 s at ~ 85 dB SPL (measured at 50 cm from the loudspeaker) [25]. Male concave-eared torrent frogs were placed 1.7 m in front of the loudspeaker and held in place by placing a transparent container (L × W × H: 27 × 15 × 17 cm) over them; this container did not impede sound transmission. The male was allowed to rest inside the container for at least 5 min. When the male frog was calm, the container was lifted, followed by the immediate playback of the stimuli (see Additional file 2: Supplementary Video S1). The vocal behaviors of male frogs were recorded with a camera (Sony HF M40, Tokyo, Japan). A microphone was attached via the camcorder's audio input connector to enable the recording of male calls; these calls were subsequently extracted from the recording with Adobe Audition 14. 2.0.34 (Adobe Inc., California, USA). Each experiment lasted from the lifting of the container until the time that the male frog approached within 10 cm of the loudspeaker. Phonotaxis tests were considered successful if male frogs approached within 10 cm of the loudspeaker [17]. Upon completion of all experiments, the frogs were released back into the wild.

### Data collection

**Fig. 2:**
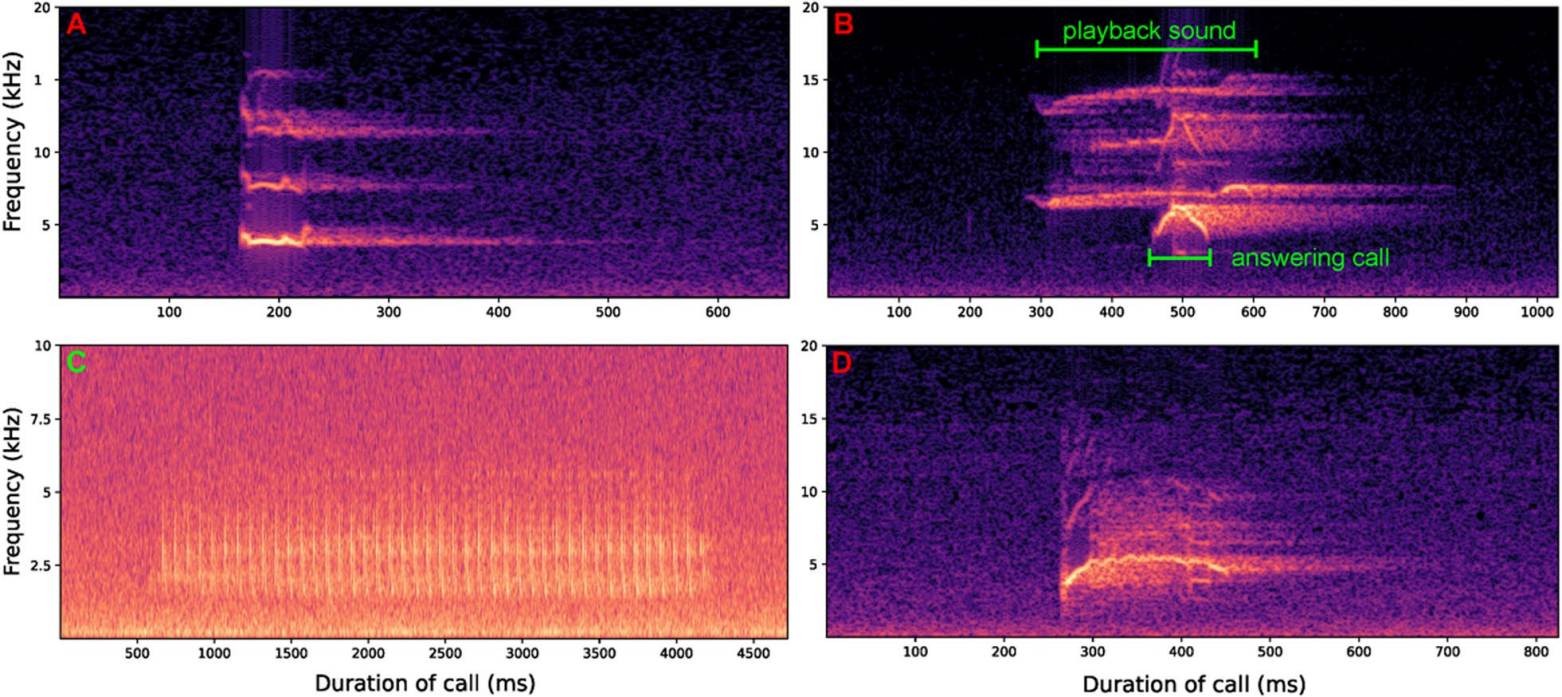
Acoustic spectra of call types produced by male frogs in the phonotaxis experiment. **A** represent short calls; **B** depicts an answering call produced by a male frog in response to a stimulus call. **C** staccato call; **D** meow. The long green bar indicates the duration of the stimulus call, and the short green bar indicates the duration of the male call elicited in response

We recorded the call type that each male produced as both the first call (i.e., the first call of the male frog after beginning the playback experiment) and the last call (i.e., the call made by a male frog when approaching to within 10 cm of the speaker) to determine whether the vocal behaviors of male frogs were influenced by the distance between males and females in the field. Different call stimuli can result in varying phonotaxis durations; hence, we conducted a statistical analysis of the latency of male frogs to approach within 10 cm of the loudspeakers. Additionally, we documented the frequency of each call type emitted by individual male frogs during the phonotaxis experiment to analyze the impact of various NLP components on vocalization behavior. In summary, we recorded 7 variables for the phonotaxis experiments: (1) the number of short calls produced (C-Sc), (2) the number of “meow” calls produced (C-Mw), (3) the number of staccato calls produced (C-St), and (4) the number of answering calls produced (C-Ac, Fig. 2), (5) time to approach within 10 cm of the loudspeaker (T-Sp), (6) call type of the first call, and (7) call type of the last call. The above data were extracted with Adobe Audition 14.2.0.34 and Adobe Premiere Pro 15.4.1 (Adobe Inc., California, USA).

### Data analysis

All the statistical analyses were performed using R for Mac OS 13.4 version 4.3.0. Since the stimulus calls vary in frequency and duration, we conducted multiple comparisons using the “*emmeans*” package to assess whether the effects of calls containing NLP components on the male frog's vocal behavior, within the same NLP components, are influenced by changes in frequency and duration. Subsequently, we applied Holm‒Bonferroni correction to adjust the *P* values for multiple comparisons. The results revealed no significant differences in the vocal behavior of concave-eared torrent frogs in response to stimulus calls that contained the same NLP components (see Additional file 1: Table S1).

We used generalized linear mixed models (GLMMs) for primary analyses; these included the NLP component as a fixed term and the call stimulus as a random effect. Due to variation in the latency of male frogs to approach within a 10 cm of the speaker, we include the log-transformed T-Sp as a fixed factor. We employed the “*glmmTMB*” package to fit GLMMs using a Poisson distribution to analyze the variable C-Mw. Additionally, for the variables C-Sc, C-St, and C-Ac, we utilized the same package to fit a GLMM with a negative binomial distribution and a log link function since a preliminary analysis using the Poisson distribution for the error term indicated a substantial overdispersion in the dataset (checked using the package “*DHARMa*”). We used likelihood ratio tests to calculate the significance of each fixed term. If there was a significant impact of NLP on vocal behavior, we employed Wilcoxon tests to further explore whether there were differences in C-Sc, C-Mw, C-St, and C-Ac produced in response to different NLP components. Finally, we used a chi-square test to examine which call types male frogs preferred to produce as the first calls and last calls, as well as to identify potential differences between the first calls and last calls.

## Results

**Fig. 3:**
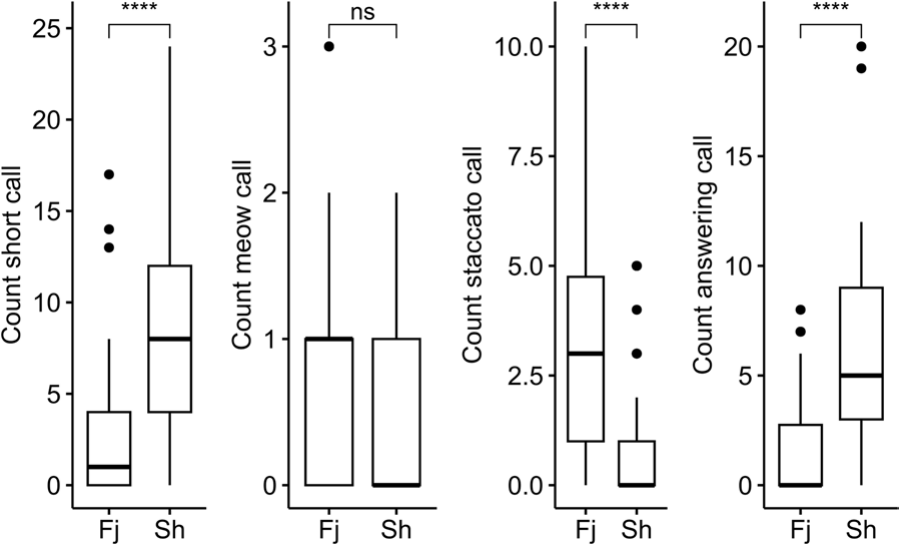
Comparison of NLP components on male concave-eared torrent frog vocal behavior. ****P < 0.0001; *ns* not significant

Male concave-eared torrent frogs demonstrated phonotaxis in playback experiments; specifically, out of the 120 males tested, 118 approached within 10 cm of the loudspeaker. In reponse to the 12 stimulus calls, male frogs emitted the following three types of calls: “meow” calls, short calls, and staccato calls [3]. The NLP components had no significant effect on C-Mw, whereas they had significant effects on C-Sc, C-St, and C-Ac (generalized linear mixed models for the C-Mw, C-Sc, C-St and C-Ac, respectively: ${\upchi }_{1}^{2}$ = 1.14, *P* = 0.29; ${\upchi }_{1}^{2}$ = 25.97, *P* < 0.001; ${\upchi }_{1}^{2}$ = 19.40, *P* < 0.001; ${\upchi }_{1}^{2}$ = 24.53, *P* < 0.001). The male frogs exhibited significant differences in C-Sc, C-St, and C-Ac in response to vocal stimuli with different NLP components, whereas C-Mw was not affected (Wilcoxon tests for C-Sc, C-St, C-Ac and C-Mw, respectively: W = 592, *P* < 0.001; W = 2796, *P* < 0.001; W = 578.5, *P* < 0.001; W = 1911, *P* = 0.30) (Fig. 3).

**Fig. 4:**
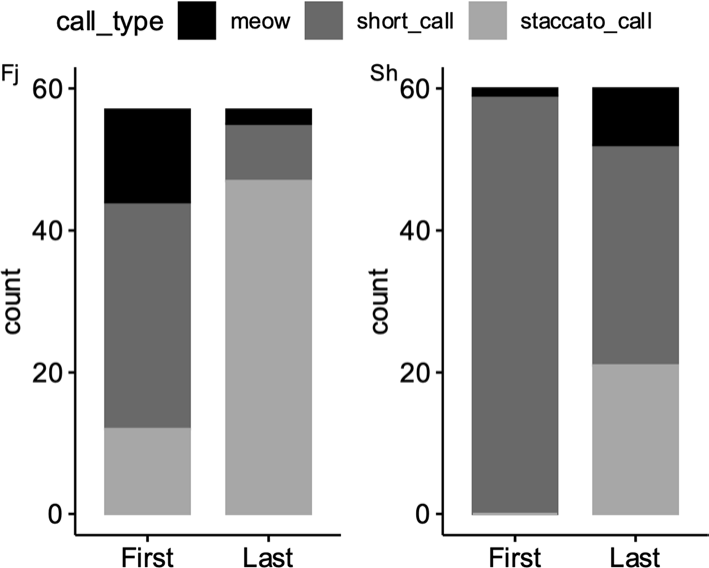
Number of male frogs that choose meows, short calls, or staccato calls as their first calls and last calls under different NLP component stimuli. Chi-square test: First_Fj_, ${\upchi }_{2}^{2}$ = 13.37, *P* = 0.001; Last_Fj_ χ^2^ = 62.84, *P* < 0.001; First_Sh_, ${\upchi }_{2}^{2}$ = 114.1, *P* = 0.001; Last_Sh_ ${\upchi }_{2}^{2}$ = 13.3, *P* = 0.001

In response to female calls containing different NLP components in calls, significant differences were observed in the first calls and last calls produced by male concave-eared torrent frogs, both in comparisons between calls containing the same NLP components and between calls containing different NLP components (Table 1). In response to the Fj component stimulation, male frogs tend to emit short calls as their first calls, while they tended to produce staccato calls as their last calls (i.e., when approaching within 10 cm of the speaker) (Fig. 4). In response to calls containing Sh components, male frogs tended to emit short calls as their first calls and as their last calls (when approaching within 10 cm of the speaker) (Fig. 4).

**Table 1:** The comparative analysis between the first calls and the last calls across different NLP components

	${\upchi }^{2}$	df	*P*
First_Fj_–Last_Fj_	43.23	1	< 0.001
First_sh_–Last_sh_	35.16	1	< 0.001
First_Fj_–First_Sh_	30.24	1	< 0.001
Last_Fj_–Last_Sh_	27.05	1	< 0.001

## Discussion

Nonlinear phenomena (NLP) are widely reported in animal vocalizations, often generating harsh or rough sounds [27]. Several hypotheses have been proposed for the use of NLP in anurans: to attract attention [17], prevent habituation [3] or serve as individual signatures [4]. The results of the present study showed that female concave-eared torrent frogs appear to utilize different NLP components in courtship calls to affect the vocal behaviors of males. Specifically, according to our results, female calls with frequency jumps elicited more staccato calls (the sound produced before amplexus) from male *O. tormota*, while female calls with subharmonics elicited more short calls (Fig. 3).

In the phonotaxis experiment, no male frogs produced long calls in response to female calls with frequency jumps and subharmonics. This is attributed to the use of long calls to advertise their sexual readiness and defend their territories [28], rather than to serve a role in the process of males approaching females. The phonotaxis results indicate that the call types of male torrent concave-eared frogs are influenced by their distance from the female, in contrast to the male Guyanan golden rocket frogs (*Anomaloglossus beebei*) [29], whose call types (89% of courtship calls contain NLP; 5% of aggressive calls contain NLP) are affected by the number or proximity of competitors. Similarly, in crickets, males produce a long-distance courtship call to broadcast to distant females [30] and then switch to quieter short-range courtship calls once they come into physical contact with a female. At the beginning and end of the phonotaxis experiment in the present study, the call types produced by males also differed significantly (i.e., the first and last calls produced in the phonotaxis experiment significantly differed) (Fig. 4), suggesting that the vocalizations of male *O. tormota* are affected by their distance from a female.

This study showed that a sudden frequency change (i.e., frequency jump) in a female call elicited a greater number of staccato calls from males. Both field and indoor amplexus experiments revealed that male frogs typically emitted staccato calls before engaging in amplexus. Combining these findings with the results of this experiment, we propose that female frog calls containing Fj components may elicit a stronger amplexus response in males (see Additional file 3: Video S2). In terms of electrophysiology, we hypothesize that the frequency jump may be a strong stimulus reaching the auditory midbrain of the torrent concave-eared frog. This speculation is based on electrophysiological findings that suggest that different frequencies of calls elicit different potentials in the auditory midbrain of male *O. tormota* [31–33]. Namely, the male torrent concave-eared frog may exhibit abrupt potential changes in its auditory midbrain after hearing a female call containing a frequency jump. However, further research is needed to support this speculation. The results of the present study, together with those of Wu [21], imply that NLP play an important role in intersexual communication in the torrent concave-eared frog. Moreover, the present study further investigated NLP components and revealed that different NLP components have different effects on the vocalizations of the torrent concave-eared frog.

Signalers can use NLP components in their calls to convey their state, as well as to influence the behavior of conspecifics and/or heterospecifics [6, 15, 34–38]. Female koalas (*Phascolarctos cinereus*) emit sounds with relatively high levels of subharmonics when they reject male mating attempts. Surprisingly, although females signal their rejection with these calls, subharmonics attract male attention, leading males to continue to vocalize [34, 35]. The present study found that female subharmonic calls elicited male frogs to emit more short calls than staccato calls. This phenomenon is similar to that reported in koalas, as mentioned above. Since female frogs prefer smaller males and there is an inverse relationship between the snout-vent length of males and the fundamental frequency of their vocalizations [17], we speculate that the presence of subharmonics in calls of female *O. tormota* may elicit further vocalizations from male frogs, thus enabling female frogs to assess the suitability of potential males.

This study supported our hypothesis that calls of female *O. tormota* that contain frequency jumps and subharmonics elicit different vocal behavior from male frogs; the call types that males produced during the phonotaxis experiment were not emitted at random. Males preferentially produced short calls, meows, and staccato calls but not long calls. Future studies should examine the effect of NLP components in male calls on the vocal behaviors of female frogs and investigate how different NLP components affect competitive vocal behaviors among male frogs. These findings may help us to better understand the evolution of calls containing NLP components, and the benefits of such calls for the reproductive behavior of anurans.

### Supplementary Information

**Additional file 1**. Supplementary Materials.

**Additional file 2**. Supplementary video S1.

**Additional file 3**. Supplementary video S2.

## Data Availability

The datasets used during the current study are available online, https://doi.org/10.5061/dryad.xpnvx0kjr.
