# Supplementary material for: Frequency jumps and subharmonic components in calls of female Odorrana tormota differentially affect the vocal behaviors of male frogs
Source: Front Zool. 2023 Dec 8;20:39. doi: 10.1186/s12983-023-00517-9 (PMC10704646; doi:10.1186/s12983-023-00517-9)
Supplement: Supplementary file 1 — Additional file 1. Supplementary Materials. [file 12983_2023_517_MOESM1_ESM.docx]

**Supplementary Material**s

**
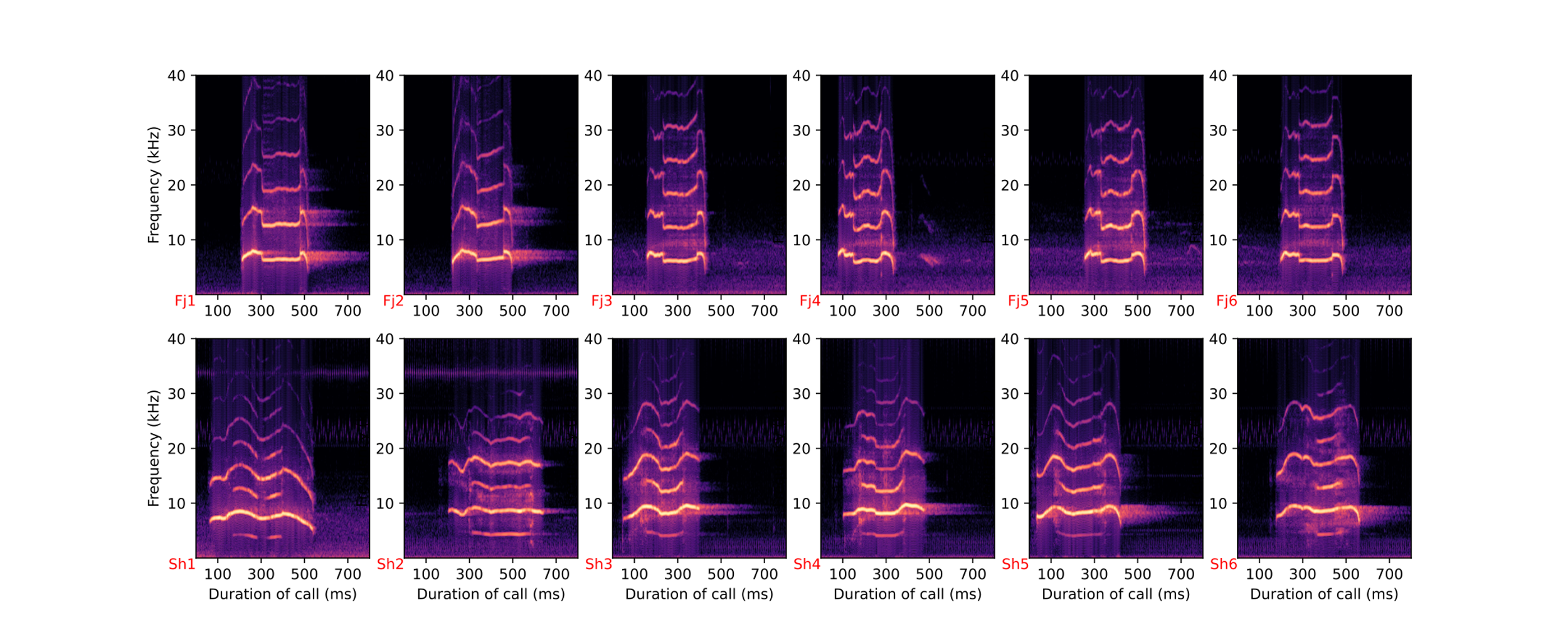
**

Figure S1: The spectrogram of 12 stimulus calls utilized in the playback experiment

Table S1: Multiple comparisons of the calling behavior of male frogs in response to the same components of NLP vocalizations, with *p* values adjusted

| contrast | count_short_call | count_meow_call | count_answer_short_call | count_staccato_call | reaching_time |
| --- | --- | --- | --- | --- | --- |
| Fj1-Fj2 | 1 | 1 | 1 | 1 | 1 |
| Fj1-Fj3 | 0.3075 | 1 | 1 | 1 | 1 |
| Fj1-Fj4 | 1 | 1 | 1 | 1 | 1 |
| Fj1-Fj5 | 1 | 1 | 1 | 1 | 1 |
| Fj1-Fj6 | 1 | 1 | 1 | 1 | 1 |
| Fj2-Fj3 | 1 | 1 | 1 | 1 | 1 |
| Fj2-Fj4 | 1 | 1 | 1 | 1 | 1 |
| Fj2-Fj5 | 1 | 1 | 1 | 0.9086 | 1 |
| Fj2-Fj6 | 1 | 1 | 1 | 1 | 1 |
| Fj3-Fj4 | 0.1061 | 1 | 1 | 1 | 1 |
| Fj3-Fj5 | 0.1675 | 1 | 1 | 1 | 1 |
| Fj3-Fj6 | 0.1943 | 1 | 0.1828 | 1 | 1 |
| Fj4-Fj5 | 1 | 1 | 1 | 0.8367 | 1 |
| Fj4-Fj6 | 1 | 1 | 1 | 1 | 1 |
| Fj5-Fj6 | 1 | 1 | 1 | 1 | 1 |
| Sh1-Sh2 | 1 | 1 | 1 | 1 | 1 |
| Sh1-Sh3 | 1 | 1 | 1 | 1 | 1 |
| Sh1-Sh4 | 1 | 1 | 1 | 1 | 1 |
| Sh1-Sh5 | 1 | 1 | 1 | 1 | 1 |
| Sh1-Sh6 | 1 | 1 | 1 | 1 | 1 |
| Sh2-Sh3 | 1 | 1 | 1 | 1 | 1 |
| Sh2-Sh4 | 1 | 1 | 0.5080 | 1 | 1 |
| Sh2-Sh5 | 1 | 1 | 1 | 1 | 1 |
| Sh2-Sh6 | 1 | 1 | 1 | 1 | 1 |
| Sh3-Sh4 | 1 | 1 | 1 | 1 | 1 |
| Sh3-Sh5 | 1 | 1 | 1 | 1 | 1 |
| Sh3-Sh6 | 1 | 1 | 1 | 1 | 1 |
| Sh4-Sh5 | 1 | 1 | 1 | 1 | 1 |
| Sh4-Sh6 | 1 | 1 | 0.4665 | 1 | 1 |
| Sh5-Sh6 | 1 | 1 | 1 | 1 | 1 |
